# Lifestyle empowerment for Alzheimer’s prevention prescribed by physicians: Methods and adaptations to COVID-19

**DOI:** 10.1016/j.cct.2024.107729

**Published:** 2024-11-02

**Authors:** Amanda N. Szabo-Reed, Amber Watts, Eric D. Vidoni, Jonathan Mahnken, Angela Van Sciver, Katrina Finley, Jonathan Clutton, Rachel Holden, Mickeal N. Key, Jeffery M. Burns

**Affiliations:** a Physical Activity & Weight Management, University of Kansas Medical Center, 3901 Rainbow Boulevard, Kansas City, KS 66160, USA; b Department of Psychology, University of Kansas, 1415 Jayhawk Boulevard, Lawrence, KS 66045, USA; c University of Kansas Alzheimer’s Disease Research Center, 4350 Shawnee Mission Parkway, Fairway, KS 66205, USA; d Department of Biostatistics and Data Science, University of Kansas Medical Center, 3901 Rainbow Boulevard, Kansas City, KS 66160

**Keywords:** Exercise, Physical activity, Healthy lifestyle, Intervention, RCT, Health care delivery, Exercise prescription, Cardiorespiratory fitness, Recruitment

## Abstract

The health care system is insufficiently capitalizing on the benefits of physical exercise in America’s aging population. Few tools exist to help clinicians incorporate physical activity into their clinical care, and barriers limit older adults from initiating and maintaining exercise programs. The Lifestyle Empowerment for Alzheimer’s Prevention (LEAP! Rx) Program has been designed to support providers and participants in lifestyle change. LEAP! Rx uses two forms of participant enrollment: physician referrals through electronic health records and self-referrals to test the efficacy of delivering a community-based exercise and healthy lifestyle program to older adults. After referral into the program, participants are randomized to receive the LEAP! Rx Program or are placed in a standard-of-care group to receive the program later. The LEAP! Rx program consists of a personalized and structured exercise program, lifestyle education, and mobile health monitoring. This includes a 12-week Empowerment phase with coaching and supervised exercise training, followed by a 40-week Lifestyle phase with intermittent supervised exercise and coaching. Lifestyle education includes monthly, evidence-based classes on optimal aging. The evaluation of LEAP! Rx focuses on 1) the assessment of implementation and scalability of the LEAP!Rx Program for clinicians and patients 2) the effect of the LEAP! Rx Program on cardiorespiratory fitness, 3) the impact of the LEAP! Rx Program on secondary intervention outcome measures of chronic disease risk factors, including insulin resistance, body composition, and lipids. If successful, this study’s findings could advance future healthcare practices, providing a new and practical approach to aging and chronic disease prevention.

## 1. Background

The unprecedented growth of the elderly population underscores the need for innovation in promoting health in America’s aging population. [1] Americans live longer than ever. [2] Approximately 20% of the population is 65 years and older, and this age group will continue to grow. [3] Aging brings increased cognitive and physical decline with dramatic rises in the risk of cardiovascular disease, stroke, diabetes, and Alzheimer’s disease (AD). [1] There is strong evidence that lifestyle interventions can modify these risks. [4]

### 1.1. Lifestyle interventions represent promising prevention strategies

Ongoing research suggests that modifying lifestyle behaviors represents an essential strategy for preventing age-related chronic disease. [4] Recent research suggests exercise also benefits the brain. [5–9] Increased cardiorespiratory fitness in early Alzheimer’s disease (AD) is associated with increased whole-brain volume, [5] most strongly in regions relevant to AD (the hippocampus and parietal lobe). [6] Longitudinal studies suggest a decline in cardiorespiratory fitness is associated with brain atrophy in early AD and cognitively normal individuals. [7] Insulin levels are also associated with cognitive performance and brain volume. [10] Specifically, insulin is known to regulate glucose metabolism, support cognition, enhance the outgrowth of neurons, as well as influence neurotrophic factors. [11] Thus, insulin regulation through physical activity is important for a healthy brain again and AD prevention. [12] Therefore, finding methods in which physical activity can be encouraged and disseminated within the community is not only important for preventing age-related systemic chronic disease but neurological ones as well.

### 1.2. Public health efforts currently fall short

There has been little systematic progress in translating lifestyle intervention research into meaningful gains in population health. While lab-based trials demonstrate efficacy in highly controlled environments, they are often impractical when translated to the broader population. Many clinicians recommend increased physical activity to their patients. This recommendation, however, lacks specificity [13]. Numerous barriers conspire against older adults to limit physical activity, including fear of injury or pain, self-motivation, lack of time, enjoyment of PA, social support, neighborhood conditions, weather, expense, accessibility and convenience of classes and facilities, and characteristics related to self-efficacy, such as confidence, expectations, perceived support, and affective responses associated with PA. [14,15] In addition, recommendations from clinicians are often singular events with little follow-up. The current approach of education and encouragement alone is insufficient: up to 25% of all adults in the U.S. are entirely sedentary, and more than half are insufficiently active to achieve health benefits [16,17], with physical activity levels declining through adulthood to low levels in older adults, [17–19] exacerbating the risk of functional decline. [20] Therefore, we must provide clinicians with specific, practical tools to help their patients initiate and sustain healthy lifestyle behaviors. This requires innovation in optimizing the delivery of lifestyle-based interventions to the public. A critical gap exists for patients to navigate between the clinician’s office and the resources to support a healthier lifestyle (i.e., fitness centers, trainers, dietitians). We have designed the LEAP! Rx Program to fill that gap, building on documented success when clinicians specifically prescribe exercise. [21–24]

### 1.3. Importance of an individualized, community-based approach

LEAP! Rx is a flexible approach using community-based fitness resources to help individuals overcome barriers such as motivation, access, and support. The LEAP! Rx Program leverages the patient’s clinician, familiar institutions, a supervised exercise prescription, and monitoring as entry points to initiate and sustain habitual physical activity. The program relies on three design features to encourage sustained changes in health behavior: individualization, feedback, and accessibility. Personal LEAP! Rx Coaches trained in motivational interviewing assess readiness for change [25] and identify individual motivations, goals, and barriers to sustained physical activity. Feedback is essential in supporting the intervention through self-monitoring physical activity with feedback loops to LEAP! Rx Coaches and clinicians. Accessibility is optimized through community-based fitness centers that provide ready expertise and infrastructure at low cost in a community-based setting close to home.

### 1.4. Summary

**Fig. 1.**
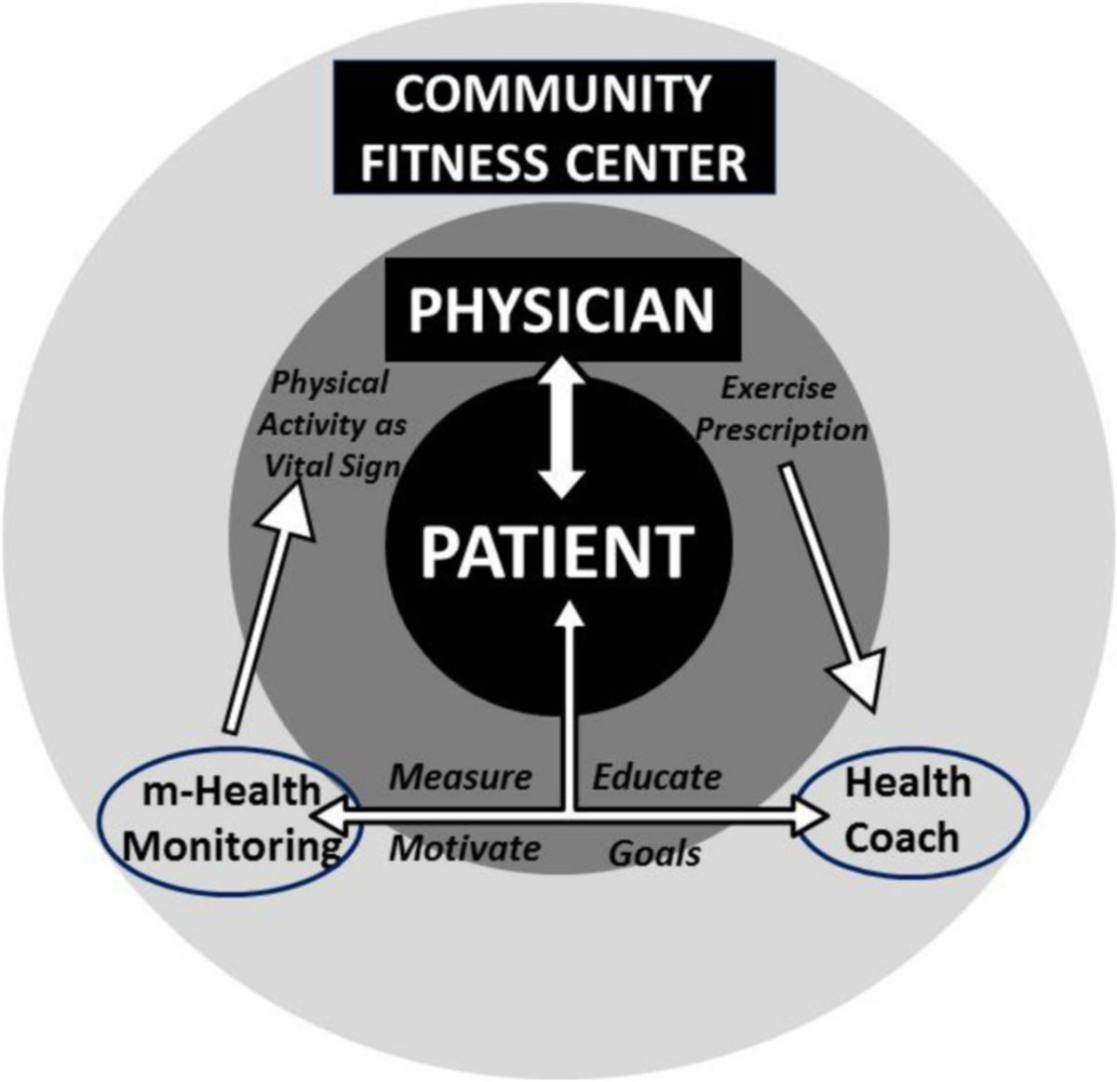
The LEAP! Rx Program supports the clinician-patient relationship. The clinician prescribes the program of coaching, training, and monitoring in a community-based fitness center that feeds easily-digested metrics back to the clinician to encourage long-term compliance.

Currently, the healthcare system is insufficiently capitalizing on the potential of lifestyle changes to reduce the population burden of chronic disease in older adults. Simply encouraging older adults to adopt healthier lifestyles is not enough. Innovation is needed to develop interventions to maximize successful aging through strategies to sustain older adults’ health and function. [26] There is a critical need to translate the latest evidence-based approaches into broad public health benefits. The primary purpose of this study is to test a framework for clinicians to engage their patients in healthy behaviors and sustained lifestyle changes following public health recommendations (see Fig. 1). The proposed framework targets important health outcomes in older adults on multiple levels:

1. To healthcare providers who need practical tools to promote prevention strategies in older adults;
2. To patients who need guidance on evidence-based strategies to maintain optimal health through proven programs that help initiate and sustain behavior changes;
3. To healthcare systems needing proven options for preventative health programs and guidance on leveraging them (i.e., referral process, links to community-based programs).

If this approach reduces the burden of chronic disease, it could have significant public health and economic consequences in the field of cognitive aging and AD. [27,28] Thus, optimizing lifestyle strategies to maximize their effect will likely translate into sizeable public health gains.

### 1.5. Hypotheses

The goals of this study are:

1. Assess the implementation and scalability of the LEAP!Rx Program for clinicians and patients, we will test the efficacy of an exercise and healthy lifestyle program using a unique referral method embedded in the electronic health record (EHR) compared to the standard of care.
2. Determine the effect of the LEAP! Rx Program on cardiorespiratory fitness. We hypothesize that the LEAP! Rx Program will increase and maintain cardiorespiratory fitness (peak oxygen consumption [VO_2peak_]) at both 12 weeks (after the initiation phase) and 52 weeks (after the maintenance phase).
3. Test the effect of the LEAP! Rx Program on outcome measures of chronic disease risk factors, including insulin resistance, body composition, and lipids. We hypothesize that LEAP! Rx Program will improve an individual’s metabolic profile with measurable benefits in specific secondary outcome measures of HOMA2, fat mass, lean mass, and cholesterol (total, LDL, HDL).

## 2. Methods

**Fig. 2.**
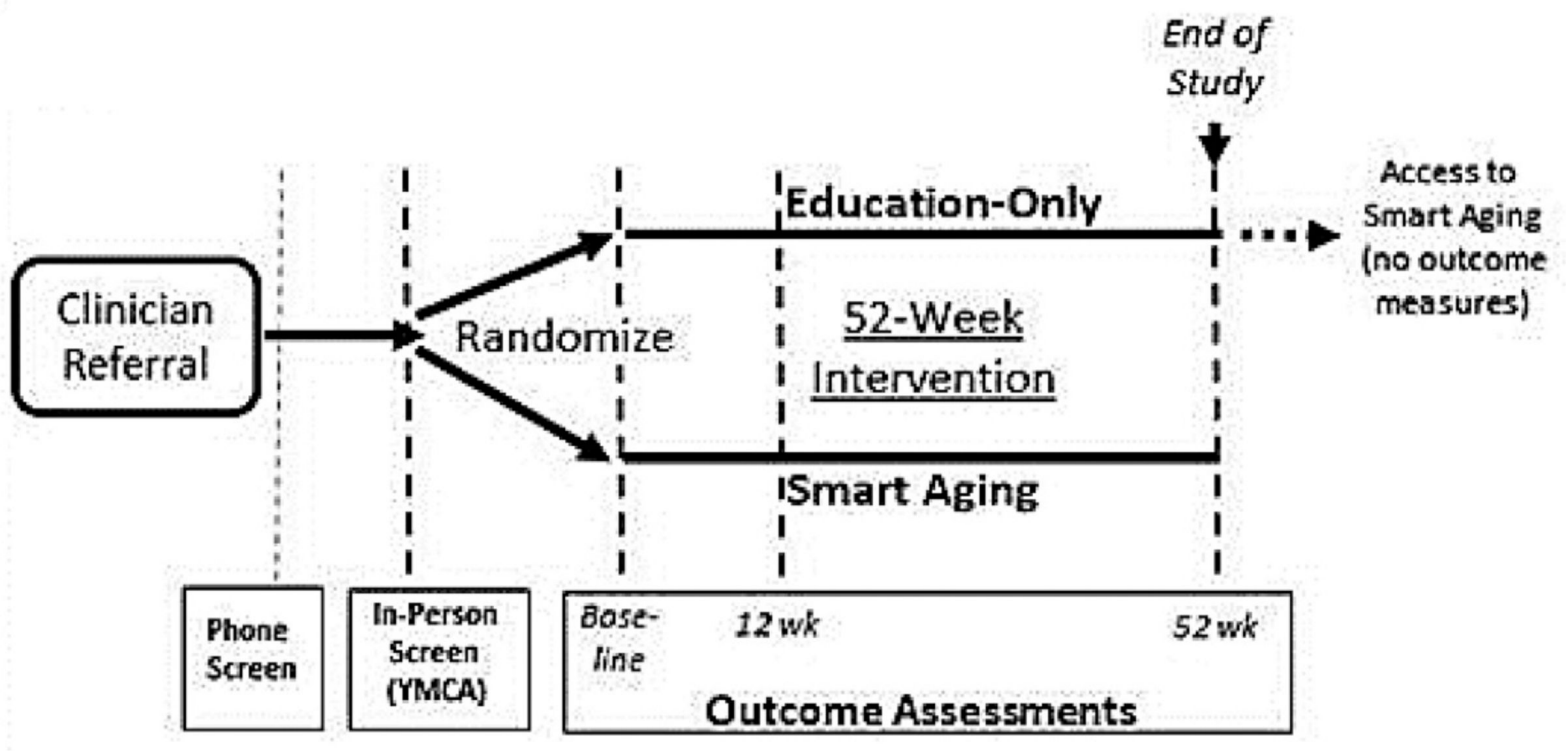
Study flow.

Our goal is to randomize two hundred twenty participants to the LEAP! Rx Program vs. a standard of care control group (1:1 ratio, Fig. 2). The standard of care group receives educational materials at entry – mimicking the current standard of care for encouraging health behavior change – and provides outcome measures parallel to those of the active intervention group. After completion of the study, this group has access to the LEAP! Rx Program (to enhance recruitment efforts; no outcomes measured).

### 2.1. Inclusion/exclusion

The inclusion/exclusion criteria (Table 1) are intentionally broad to provide a generalizable cohort of older adults and encourage clinician referral of participants who the clinician believes would benefit from increased physical activity. Participants are clinician-referred or self-referred, ambulatory, underactive, and 65 years and older without a recent history of stroke or coronary artery disease.

### 2.2. Referral and recruitment process

#### 2.2.1. Referral process

We recruit participants by two modes: direct referral from clinicians (i.e., physicians, nurse practitioners) of the hospital or the general population via typical recruitment methods such as advertisement, flyers, and in-person talks. Our referral process for clinicians is embedded in the EHR as a “prescription” so that the LEAP! Rx Program is ordered as part of a seamless clinical workflow. Workflow development included 1) identifying precise, computable phenotypes to identify potential participants, 2) flagging these potentially eligible participants for providers (“best practice alert”), and 3) routing the prescription from the health system through the study team with minimal delay, and on to activation for enrollment at the YMCA. The ideal flow would be direct from provider to community support. However, for research rigor, the electronic prescription or recruitment division referral to the study team allows initial contact to determine eligibility and outcome test scheduling. A clinician or physician clearance to exercise is required for self-referred participants.

#### 2.2.2. Clinician role

Clinicians “prescribed” the program to potentially eligible patients via an electronic prescription, just like other orders in the EHR. Evidence suggests that a brief 3–4 min office discussion is meaningful without adding a significant burden and can aid in program recruitment and adoption. [30]

#### 2.2.3. Phone screening

KU study staff contact participation candidates to assess eligibility, obtain permission to examine their health records and perform a Telephone Assessment of Physical Activity [29] to ensure participants are sedentary or underactive.

#### 2.2.4. Enrollment and randomization

After completing the phone screen, eligible participants are given a standard set of informational materials on the study. If the potential participant is interested, staff schedule the baseline visit. Baseline testing is scheduled with a goal of completion within a two-week window. Upon completing baseline testing, the study coordinator randomizes participants to either the active intervention or the standard of care 1-year wait-list control group (1:1 ratio). The study statistician developed the randomization table with random block sizes in Statistical Analysis Software (SAS©, SAS Institute Inc., version 9.13) and uploaded to the data capture system, REDCap, to securely allocate participants to the treatment arm.

#### 2.2.5. Blinding

Psychometrists and exercise physiologists who perform assessments are blinded to intervention groups. The primary investigator is unblinded to perform safety assessments and address safety concerns or adverse events but is not involved in acquiring outcome measures and does not have access to the outcome measures in REDCap.

### 2.3. Control group: Standard of care

The standard of care group has been designed to represent the current standard of clinical care. Individuals randomized to this group are only given educational information on the benefits of a healthy lifestyle at baseline but are not discouraged from exercising independently. However, we ask that they agree not to join a YMCA or hire a personal trainer during this time. Current data suggest that education only does not result in significant and sustained physical activity changes. [21,31,32]

Participants randomized to the standard-of-care group are informed they have been randomized to a 52-week “waiting list,” during which they complete all outcome measures parallel to the active intervention group. All participants (both active and standard of care) receive the same educational materials at the enrollment visit. After completing outcome assessments at 52 weeks, the standard-of-care participants are offered access to the full LEAP! Rx Program at no cost, including membership and coaching fees, to enhance recruitment and minimize dropout from this group. Outcome assessments are not repeated during their participation in the LEAP! Rx Program.

Cross-over does not threaten internal validity as the study is designed to assess the value of the LEAP! Rx Program over and above the effects of standard of care (i.e., education only). Control participants can exercise independently (though no structured assistance is provided for this comparison group).

### 2.4. Intervention group: LEAP! Rx program

The LEAP! Rx Program is packaged to be delivered at any fitness center. For this study, the investigators partnered with local YMCA sites. The LEAP! Rx Program represents a “package” consisting of:

1. A dedicated LEAP! Rx Coach (i.e., personal trainer)
2. Mobile health objective monitoring of daily physical activity
3. Smart Aging Educational Curriculum
4. Group exercise opportunities

Within this program, participants are encouraged to complete the current public health recommendations for physical activity, which includes 150 min of moderate to vigorous physical activity and two days per week of resistance training/balance training. [33,34] Details related to exercise prescription, including frequency, intensity, time, and type of exercise recommended for LEAP!Rx program can be found in Supplemental Material 1. [35,36]

### 2.5. Response to the COVID-19 pandemic

In March 2020, exercise facilities were closed in response to the COVID-19 pandemic. Many facilities remained closed until the end of May 2020. During this time, participants continued meeting with their LEAP! Rx Coach virtually over Zoom to complete exercise sessions at home. In addition, in-person education curriculum sessions were discontinued and only offered online. After May 2020, those participants who were comfortable and interested in resuming in-person exercise did.

### 2.6. Study events

#### 2.6.1. Overview

Physiologic adaptations to the LEAP! Rx Program are assessed with cardiorespiratory fitness testing, physical function assessment, body composition measures, laboratory tests, health surveys, and cognitive testing (Table 2). Study assessments are completed in one appointment visit lasting approximately two hours. (See Table 3.)

#### 2.6.2. Baseline, week 12, and week 52 outcome assessments

Baseline, 12-week, and 52-week outcome assessments are identical and included in Table 2. Consented participants had fasting phlebotomy and were provided with a snack. Trained research staff collect demographic information, health history, and medications at baseline (and update at follow-up visits). Blood pressure (measured three times in a sitting position), height, and weight were assessed each time. Trained staff complete DEXA scanning and cardiorespiratory fitness testing with graded maximal exercise testing. Participants complete less than 2 h of surveys (NIH Toolbox Emotion, diet survey, skin carotenoid spectroscopy (Veggie Meter®, Longevity Link Corp), and brief cognitive evaluation (NIH Toolbox Cognitive). These procedures take approximately two hours to complete in a single visit. An accelerometer is placed on the participant at the first visit and removed at the second visit unless extenuating circumstances arise. In this case, the accelerometer is mailed with wear instructions.

#### 2.6.3. Cardiorespiratory fitness

Our intervention outcome is cardiorespiratory fitness as measured by peak oxygen consumption (VO_2 peak_) during treadmill testing at baseline, 12 and 52 weeks. This outcome will test our hypothesis that the LEAP! Rx Program will increase VO_2 peak_ after the intensive Empowerment phase (12 weeks) and remain increased over baseline during the Lifestyle phase (52 weeks).

VO_2 peak_ is measured during the graded treadmill test to volitional exhaustion or following American College of Sports Medicine (ACSM) guidelines [37]. The test is led by an exercise physiologist and supervised by a clinician using a modified Bruce protocol. [38] Participants are attached to a 12‑lead electrocardiograph and wear a non-rebreathing facemask to assess heart rate, blood pressure, and expired air (TrueOne, Parvomedics, Sandy UT). VO_2 peak_ is the highest observed value of oxygen utilization during the test. [39,40]

#### 2.6.4. Physical activity measures

##### Accelerometry:

We use wrist-worn triaxial accelerometers (Actigraph GT9X, Pensacola, FL) for three one-week periods (baseline, week 12, and week 52). We have chosen these devices based on recent literature relevant to older adults. [41–43] The accelerometer is worn on the non-dominant wrist during a consecutive 7-day period. Participants are instructed to wear the unit 24 h a day. The unit is waterproof and can be worn while bathing and swimming. Total daily activity (sum of all activity during waking hours), the intensity of daily activity (divide total activity by the time of all nonzero epochs), and the percent of the day spent in non-activity will be calculated using ActiLife software [44] including the selection of proper epochs (1 min [45]), length of activity bouts, intensity thresholds, data transformation, and missing data imputation. [46] We consider days with >10 h of wear time to be a valid day and require at least four valid days (including one weekend day) of observation. [47]

The International Physical Activity Questionnaire (IPAQ) survey is administered to assess the participant’s habitual level of physical activity. IPAQ is a 27-item measure developed to assess physical activity levels in older adults. It is a robust measure (unaffected by seasonal bias) designed to capture various physical activities in older adults. This instrument is a valid and reliable method to assess changes in response to interventions. [48–50]

#### 2.6.5. Physical function

After training by study staff and demonstrating competence, the YMCA LEAP! Rx Coaches perform the 6-Minute Walk Test (6MWT), a well-known test of aerobic endurance [51] and has been shown to capture training responses. [52] The LEAP!Rx Coaches administer the 6MWT during the first intervention session at the YMCA and again at Weeks 12 and 52.

#### 2.6.6. Body composition

Participants have their body composition measured using dual-energy X-ray absorptiometry (DEXA, Lunar Prodigy, version 11.2068, Madison, WI) to determine fat-free mass, fat mass, and percent body fat. After attempting to void and removing jewelry, participants are weighed with a digital scale accurate to ±0.1 kg in a standard hospital gown.

#### 2.6.7. Laboratory measures

A fasting blood draw (~ 35 mL total) is performed and processed to generate plasma and serum for biomarker analysis. Plasma and serum are stored at −80C for future analysis of glucose, insulin, lipids, and related metabolic markers.

#### 2.6.8. Patient-centered measures

Patient experience, psychosocial outcomes, attendance and dropout rates, satisfaction, quality of life, and self-efficacy are assessed at baseline and 12 and 52 weeks.

##### NIH Toolbox Emotion Battery:

The NIH Toolbox Emotion Battery [53] includes a variety of measures that are used to assess participants’ responses to the LEAP! Rx Program. The battery consists of validated measures of General Life Satisfaction [54] and Self-Efficacy [55]. Additionally, it includes several relevant mental health measures such as Positive Affect, Sadness, Emotional Support, [56] and Perceived Stress. [55].

Health-related quality of life (HR-QOL) refers to how health impacts an individual’s ability to function and perceived well-being in physical, mental, and social domains. The Medical Outcomes Study 36-Item Short Form (SF-36) health survey is the most commonly used HR-QOL measure. The SF-36 health survey is a widely used health status questionnaire comprised of 36 items selected from a larger pool of items used by RAND in the Medical Outcomes Study (MOS). [57]

##### Dropout, adherence, and attendance:

We monitor dropout rates. We anticipate measuring adherence as the number of exercise sessions completed (center-based training, group sessions, and home-based sessions), the number of scheduled exercise training sessions completed, and the number of LEAP!Rx Coaching sessions attended. Participants maintain paper-based logs to record their home-based exercise and group exercise attendance. LEAP!Rx Coaches enter data from the paper-based logs into a central database maintained by KU staff. Additionally, attendance at scheduled LEAP! Rx education classes (monthly), exercise training sessions, and LEAP!Rx Coaching sessions are recorded by YMCA staff and entered into the central database. Attendance data is shared with Referring Clinicians.

##### Exit surveys:

We also perform a short semi-structured exit survey on all patients who complete the study (at Week 52 visit) to assess their general experience, perceived benefits, and suggestions for improvement. Participants who drop out are interviewed by phone to ascertain their reasons for dropping out, perceived barriers, suggestions for program improvement, and general feedback.

#### 2.6.9. Clinician measures

Because our overall object is to create a framework for clinical lifestyle prescription, we aim to create a clinician tool for the effortless “prescription” of the LEAP! Rx Program. In return, the clinician receives simple, easily digestible metrics on patient progress (attendance and physical activity data) to support the clinician-patient relationship.

Referral patterns are assessed per clinic type and clinician by examining the number of referrals, timing of referral, and clinical indication for referral. Examining the time and source of referrals will provide insight into adoption rates, whether program growth is organic vs. driven by our efforts to engage clinicians, and whether these adoption rates are variable across clinics.

#### 2.6.10. Other outcomes

##### NIH Toolbox Cognitive Function:

Although not a primary aim of this project due to power issues, we will collect basic cognitive function data using a brief battery derived from the NIH toolbox. [58] This information may prove useful in powering a larger future study if needed.

##### Diet intake survey:

As greater attention to one’s lifestyle may lead to changes in the composition of our participant’s diets, we assess changes in diet during the intervention using the NCI Diet History Questionnaire II as an exploratory outcome. Diet is strongly linked to diabetes, obesity, hypertension, and inflammation, while specific diets have been linked with AD and dementia outcomes. [59–61]

##### Skin carotenoids:

Skin carotenoids are measured at the baseline and final visits in a sub-sample of participants. [62] This tool uses reflection spectroscopy to measure skin carotenoid levels, which can be associated with fruit and vegetable consumption. The participant will place an index finger in the probe for 10 s while the skin tissue is measured. Three measurements are taken for an average score.

### 2.7. Data management

Study data are collected and managed using web-based, electronic data capture tools hosted on a secure, HIPAA-compliant server. Data are collected on standard source documents or via direct data entry into REDCap hosted on a secure institution server with role-based access.

### 2.8. Sample size

Our overall goal is to create a framework for clinical lifestyle prescriptions. The primary intervention measure – VO_2peak_ at both 12 and 52 weeks – is the basis for the sample size of our study. Our analysis will be an intent-to-treat comparison of active vs. control groups using a linear mixed model of repeated measures of VO_2peak_ at initial measurement, 12 weeks later, and after 52 weeks. Study power is based on a two-degree-of-freedom test of our priori-identified single primary intervention outcome measure (VO_2peak_) at two-time points. We will use a contrast matrix to test both time points simultaneously, so no multiplicity adjustment will be needed. For all analyses, the Type I error rate will be set to α = 0.05, and we will use a contrast matrix to conduct the F-test of our primary research hypothesis, which notably mimics a two-sided testing paradigm to even further protect against spurious results. Additional details are available in Supplemental Materials 2.

### 2.9. Outcomes and planned statistical analyses

This study aims to assess the implementation and scalability of the LEAP!Rx Program for clinicians and patients. Implementing the program requires both patient and clinician satisfaction. To assess the implementation and scalability of the LEAP!Rx Program for clinicians and patients. We will test the efficacy of an exercise and healthy lifestyle program using a unique referral method embedded in the electronic health record (EHR) compared to the standard of care. Patient-centered outcomes will also involve generalized linear mixed models (GLMMs) at baseline, 12, and 52-week time points. The linear mixed models (LMMs, a special case of the GLMMs) will suffice for continuous measures.

Secondarily, we hypothesize that the LEAP! Rx Program will increase and maintain cardiorespiratory fitness (peak oxygen consumption [VO_2peak_]) at both 12 weeks (after the initiation phase) and 52 weeks (after the maintenance phase; co-primary outcome measure). VO_2peak_ is a vital health outcome linked to mortality and age-related diseases. It can be viewed as a physiological “assay” to prove that our intended health behavior change (increased physical activity as measured by accelerometry and survey) induces meaningful physiological benefits. We also expect increased moderate-to-vigorous physical activity (measured by accelerometry and survey) to be responsible for VO_2peak_ benefits. The primary study measure, VO_2 peak_, will have repeated measures over time (baseline, week 12, and week 52). Thus, we will use linear mixed models (LMMs) for analysis.

Finally, we are also interested in the effects of the LEAP! Rx Program on secondary outcome measures of chronic disease risk factors, including insulin resistance, body composition, and lipids. We hypothesize that LEAP! Rx Program will improve an individual’s metabolic profile with measurable benefits in specific secondary outcome measures of HOMA2, fat mass, lean mass, and cholesterol (total, LDL, HDL). The secondary study measures for Aim 3 will be collected at baseline, 12-, and 52-week time points; thus, similar analysis methods as in Aim 1 will be used (LMMs). Additional details on statistical analysis are available in Supplemental Material 3.

## 3. Conclusion

The ultimate goal of the LEAP! Rx study aims to create a scalable and cost-effective program for clinicians and their patients that reduces the risk of chronic disease by inducing lifestyle behavior change. The LEAP! Rx program begins with and supports the clinician-patient relationship to provide clinicians with a new tool composed of 1) an evidence-based prescription [33,34], 2) a framework for delivering the prescription, and 3) metrics to assess effects on behavior. Participants are recruited through clinicians and facilitated by an electronic prescription through the EHR that seamlessly integrates with the clinician’s workflow.

We use several methods to support continued behavior maintenance in real-world settings (i.e., mobile health monitoring for real-time feedback, community-based centers, and regular follow-up) to overcome barriers to sustained behavior change. The approach of the LEAP! Rx study is designed to realign common resources to scale the program to any staffed fitness center. We draw on our YMCA’s experience scaling other interventions (Diabetes Prevention Program) and technology that will soon be ubiquitous (i.e., smartphones or wearable computing). If effective in this rigorous clinical trial, we will deliver an innovative program nationally to improve population health.

## Supplementary Material

LEAP! Rx Certification Checklist

Program Coach Manual

Participant Expectations

Supplemental Material

## Figures and Tables

**Table 1:** Inclusion/exclusion criteria.

Inclusion criteria	• Clinician-referred or self-referred • Age 65 and older • Ambulatory: walk unassisted and complete the 6-min walk test without rest • Sufficiently fluent in English to participate in study procedures • Sedentary or underactive by the Telephone Assessment of Physical Activity [29] • PI’s judgment regarding potential participants’ health status and likelihood of completing the 1-year intervention • Clinician or physician clearance to exercise
Exclusion criteria	• Clinically significant systemic or psychiatric illness that may affect safety or completion • Use of Insulin • Diagnosis of dementia; use of dementia medications (cholinesterase inhibitors, memantine) • Myocardial infarction or unstable coronary artery disease (e.g., angina, arrhythmia) in the last six months. • Cerebrovascular event (stroke or transient ischemic attack) in the last six months • Cancer diagnosis in the last two years (except non-metastatic basal or squamous cell carcinoma or cancer in remission in the absence of treatment for at least two years) • Significant pain or musculoskeletal disorder limiting the ability to participate safely. • Another member of the household enrolled in the study • Presence of a pacemaker or defibrillator

**Table 2:** Study assessments.

			Visit 1		Visit 2		Visit 3
	Referral			12-week Empowerment Phase		36-week Lifestyle Phase	
	Week - 4		Baseline		Week 12		Week 52
**Study Events**							
Chart Review -- Meds, PMH	**X**						
Phone Screen (Health Hx, TAPA)	**X**						
Consent			**X**				
Peak VO2			**X**		**X**		**X**
DEXA / Height, weight			**X**		**X**		**X**
PA assessment - accelerometry			**X**		**X**		**X**
Fasting Blood Draw			**X**		**X**		**X**
NIH Emotion, Cognitive Toolbox, PROMIS			**X**		**X**		**X**
NCI Diet History Questionnaire 2, Veggie Meter			**X**		**X**		**X**
Randomization			**X**				

**Table 3:** Outcome measures and their duration.

Outcome measure	Duration
Phlebotomy and physical function	125 mins
Blood draw / vitals	15 mins
DEXA / Ht / Wt	25 mins
VO2 assessment	40 mins
6-min walk test (YMCA)	10 mins
NIH cognitive	31 mins
Attention (Flanker)	3 mins
Episodic memory (picture sequence)	7 mins
Working memory (list sorting)	7 mins
Language (reading recognition)	7 mins
Executive function (card sort)	4 mins
Processing speed (pattern comparison)	3 mins
Accelerometer placement & instructions	<5 mins
Questionnaires/Other	85 mins
NIH emotion	22 mins
Psychological well-being	6mins
Stress and self-efficacy	4 mins
Social relationships	8mins
Negative affect	4 mins
PROMIS	6 mins
Fatigue	2 mins
Pain interference 4a	2 mins
Pain behavior 7a	2 mins
Physical activity level	7 mins
Health-related QOL (SF-36)	10 mins
NCI diet history questionnaire 2	30 mins
Veggie meter	5 mins
Exit surveys (at week 52)	5 mins

## Data Availability

No data was used for the research described in the article.

## Abbreviations:

LEAP!: Rx Program, Lifestyle Empowerment for Alzheimer’s Prevention
EHR: Electronic Health Record
m-Health: mobile-health
AD: Alzheimer’s Disease
KU ADRC: University of Kansas Alzheimer’s Disease Research Center
IPAQ: International Physical Activity Questionnaire
6MWT: 6-Minute Walk Test
DEXA: dual-energy X-ray absorptiometry
HR-QOL: Health-related quality of life
GLMMs: generalized linear mixed models
LMMs: linear mixed models
