# Supplementary material for: Lifestyle empowerment for Alzheimer’s prevention prescribed by physicians: Methods and adaptations to COVID-19
Source: Contemp Clin Trials. Author manuscript; Available in PMC 2025 Mar 24. (PMC11932157; doi:10.1016/j.cct.2024.107729)
Supplement: LEAP! Rx Certification Checklist [file NIHMS2037346-supplement-LEAP__Rx_Certification_Checklist.pdf]

KU ALZHEIMER'S DISEASE CENTER
The University of Kansas Medical Center

LEAP! Rx Certification Checklist

1) Highlights
   - **Teach** participants how to accomplish the aerobic and resistance exercise goals **independently.**
   - **150 minutes of aerobic exercise** per week at a "somewhat hard" intensity (4-5 on a modified (1-10) Borg Scale)
   - **2 resistance training sessions per week** with some understanding how to program their own sessions
   - **Teach** participants to **independently** log their own **data**

2) Personal Coaching Sessions

   Empowerment Phase – Weeks 1 – 12

   The Empowerment Phase is intended to empower older adults, who are currently underactive or sedentary, to embrace exercise slowly and gradually over 6 weeks and begin to meet physical activity and exercise guidelines. During this phase focus on training the participant how to complete the exercise and fill out the log **independently**
   - Weeks 1-6: 2 times per week
     Week 1, session 1 includes a 6-minute walk test conducted by the trainer
     Weeks 8, 10 & 12: 1 time per week
     Week 12 includes a 6-minute walk test conducted by the trainer

   Lifestyle Phase – Weeks 13 – 52

   The Lifestyle Phase is intended to assist older adults to maintain the newly adopted exercise regimen and adopt healthy lifestyle changes by attending monthly educational sessions and encouragement from you. During this phase focus on ensuring the participant is maintaining their physical activity.
   - Weeks 13 - 52: 1 time per month for one-on-one sessions with you and monthly education conducted by study staff.
     Week 52 includes a 6-minute walk test conducted by the trainer

3) Exercise Dose and Schedule

   Aerobic Exercise:

   Aerobic exercise is our primary outcome, so please empower participants to complete all the aerobic exercise minutes at the appropriate intensity level. Focus your attention on the aerobic exercise goals during one-on-one sessions, if the participant is not meeting the goal via independent sessions.
   - In weeks 1-6, duration and frequency will increase from 60 minutes per week over 3 days, to 150 minutes per week over 4-5 days (or more). Frequency will depend on participant's tolerance for the exercise. Participants will continue with aerobic exercise 150 minutes per week for weeks 7-52 (the remainder of the study). To accomplish this, you will need to **empower participants to complete aerobic exercise sessions independently, starting in week 1**. Independent aerobic exercise session minutes can be accumulated through appropriate group exercise classes, or using treadmill, elliptical, stationary bike, etc.

   - Intensity- Target Heart Rate (THR) zones are provided by study team based on V02 testing and are indicated in the participant's exercise log. THR will increase over time and a progression table will be provided to you. As a trainer, do your best to help participants reach their THR and how it relates to the *modified (1 – 10)* Borg Scale or Rating of Perceived Exertion (RPE). Participants will self-monitor their independent sessions using the using the modified RPE scale with a **goal of working in the "somewhat hard" or "hard" range (4-5). For** this reason, it is imperative the participant is well-educated on the RPE scale and what it feels like to exercise at a moderate intensity level.

Strength and Resistance Exercise:

Strength training, although important for participants to engage in, is not our primary outcome. Focus your attention on the aerobic exercise, unless the participant is meeting the aerobic goals on their own. If they are meeting the aerobic exercise goals, then begin to focus on strength training during one-on-one sessions.

- Teach participants to complete 2 resistance training sessions per week
- Minimum of 1 set of 8-12 repetitions for 8-10 exercises covering the major muscle groups; keeping in mind their past exercise experience, past injuries, personal goals and typical needs/concerns of older adults such as addressing kyphosis, foot shuffling, etc.
- Participant injuries will be included in their exercise folder. Be sure to also ask the participant about any other injuries or issues before training. Avoid overhead exercises and squats below 90 degrees for shoulder pain and hip replacements respectively.
- Participants can accomplish the strength training goals through appropriate group exercise classes, or in the weight room.

Group Exercise:

- Identify appropriate group classes for both aerobic and strength exercise from a group fitness schedule. Some participants enjoy the social aspect of group exercise more than others. If the participant enjoys group exercise, find classes that work for him or her. As the participant progresses through the program help them to find progressively challenging classes.
- The group exercise can be applied to the weekly aerobic (150 min) and resistance exercise (2x per week) goals but will not replace the Personal Coaching Sessions.
- Teach participants how to log their group exercise in their exercise log. Classes that are either all aerobic or resistance should be logged based on the amount of time they engaged in that activity in the class. Classes that have both aerobic and resistance exercise should be split based on the estimated time in the exercise log.

4) Personalized Exercise log
   - Participants are provided a personalized exercise log pre-populated with the desired frequency, intensity and duration by week. **Please instruct participants how to complete the log and teach them to complete the log on their own to record exercise completed during independent exercise session**. They will indicate whether the session was completed during a one-on-one session with you, in a group class or independently. The exercise log is our study data, so it is imperative it is fully completed.

5) Garmin Vivofit and Garmin hubs
   - Participants wear Garmin Vivofit watches that register their daily steps. These steps are sent back to their physicians. The watches sync to their phones and the Vivohubs installed at the gyms.
   - The Vivohubs register when a new participant walks into the room by showing a walking man with a bar on the screen. Once the blue bar has fully loaded, the participant's data has been synced.
   - These watches are not your responsibility. If a participant asks you for help, do your best to help them but if you can't figure it out, refer them to us.
   - Some older exercise logs have steps included. Participants do not need to fill these out.

6) Monthly Education
   - After week 4, participants attend monthly education sessions at the KU ADC. These sessions are typically held the second Tuesday of the month at 1 PM at 4350 Shawnee Mission Parkway.

7) Membership and use of the YMCA
   - Participants receive a study-paid membership. Encourage participants to complete all exercise sessions at the YMCA. If the participant wants to exercise at home or otherwise outside of the YMCA, it is permissible, but not preferred. All exercise completed by the participant, regardless of location, must be recorded on the provided exercise log, which will stay at the YMCA.

- If the participant goes on vacation or is ill for two or more consecutive weeks and does not exercise, you should adjust and reintroduce exercise gradually, to work up to their prior frequency, duration and intensity before they stopped exercising. Use best judgement.

8) Injury Prevention, Individualization, and Adverse Events (AEs)
    - A year is a long intervention. We have seen many older adults develop hip, knee, back or foot issues throughout the study. Monitor your participants for overuse and overtraining and help the adjust accordingly. Safety is of utmost concern over reaching goals of the study.
    - Treadmill is preferred method of aerobic exercise, especially during the Empowerment Phase (weeks 1-12). Participants who begin having hip, knee, back or foot issues may need to switch modalities to prevent overuse injuries. Encourage participants to notify you they are ready to try a new aerobic modality, so you can assist them on safety and use of the machine.
    - Each time you meet with the participant ask if they have experienced any Adverse Events (AEs). An AE is any illness, injury or medication change they have had. Regardless of the AE's relationship to the exercise intervention, please report all AEs to the study coordinator.

9) The Study Coordinator has explained how to conduct the intervention, monitor participants for safety, report AEs, and to contact the study team to relay important information, questions, or concerns.

____
Printed name of Study Trainer

______________________________ ____________________
Signature of Study Trainer Date

______________________________ ____________________
Signature of Study Coordinator Date

Notes and Suggestions:

____

____

____

____

____

____

____

____

____

____

____

Empowerment Phase

Week #	Supervised Sessions<br><br>Per week	Total Minutes Per Week<br><br>Aerobic Exercise	Target Heart Rate Zone<br><br>No Beta Blocker	Target Heart Rate Zone<br><br>On a Beta Blocker	Rate of Perceived Exertion<br><br>(Scale 1 -10)
1	2	60	40 – 55%	30 – 45%	3 - 4
2	2	75	40 – 55%	30 – 45%	3 - 4
3	2	96	40 – 55%	30 – 45%	3 - 4
4	2	118	45 – 60%	35 – 50%	3 - 5
5	2	139	45 – 60%	35 – 50%	3 - 5
6	2	150	45 – 60%	35 – 50%	3 - 5
7	0	150	45 – 60%	35 – 50%	3 - 5
8	1	150	45 – 60%	35 – 50%	3 - 5
9	0	150	45 – 60%	35 – 50%	3 - 5
10	1	150	50 – 65%	40 – 55%	4 - 5
11	0	150	50 – 65%	40 – 55%	4 - 5
12	1	150	50 – 65%	40 – 55%	4 - 5

Lifestyle Phase

Week #	Supervised Sessions<br><br>Per month	Minutes Aerobic Exercise<br><br>Per Week	Target Heart Rate Zone<br><br>No Beta Blocker	Target Heart Rate Zone<br><br>On a Beta Blocker	RPE<br>(Scale 1 -10)
13 - 52	1	150	50 – 65%	40 – 55%	4 - 5

School of Medicine | Department of Neurology
Mail Stop 6002 | 3901 Rainbow Boulevard | Kansas City, KS 66160 | 913-588-0555 | 913-945-5035 (fax)
