# Supplementary material for: Lifestyle empowerment for Alzheimer’s prevention prescribed by physicians: Methods and adaptations to COVID-19
Source: Contemp Clin Trials. Author manuscript; Available in PMC 2025 Mar 24. (PMC11932157; doi:10.1016/j.cct.2024.107729)
Supplement: Program Coach Manual [file NIHMS2037346-supplement-Program_Coach_Manual.pdf]

LEAP!
Rx
LIFESTYLE EMPOWERMENT FOR ALZHEIMER'S PREVENTION

LEAP! Rx Program Coach Manual

KU
ALZHEIMER'S DISEASE CENTER
The University of Kansas Medical Center

The purpose of the LEAP! Rx Program:

The purpose of the study is to learn whether the LEAP! Rx Program, which consists of exercise and education on healthy lifestyle choices, can **help older adults at risk for Alzheimer's disease, meet current physical activity guidelines, improve aerobic fitness,** and other physical health measures, such as cholesterol and blood sugar.

Alzheimer's Disease (AD) and Modifiable Risk Factors:

****Note: The number one risk factor for AD is AGE!***

The modifiable risk factors for Alzheimer's disease include:

- Coronary Artery Disease (heart disease)
- Diabetes (metabolic disease)
- Hypertension (high blood pressure)
- Hyperlipidemia (high cholesterol)
- Overweight and Obesity
- Lack of physical activity and social engagement
- Poor diet and sleep habits
- Depression

Exercise has been shown to be a useful tool to improve health and reduce risk for conditions such as heart disease, high blood pressure, high cholesterol, obesity, and diabetes. The KU ADC feels strongly that, "what is good for the heart, is good for the brain." One reason we believe this is because the modifiable risk factors for AD (listed above) are many of the same conditions that can be modified with exercise, and exercise is good for the heart.

All participants are referred by a KU physician, and the LEAP! Rx Program provides feedback to physicians on their patient's progress.

The LEAP! Rx Program is one-year and includes educational curriculum to support the exercise program to teach healthy choices and promote lifestyle changes to further modify risk.

****Note: The primary outcome for the study is to improve aerobic fitness. Although the program incorporates 2 days per week of strength training, we are not testing strength in the clinic. Please focus on aerobic exercise first, then strength training.***

The LEAP! Rx Program Consists of 2 Phases:

1. **The Empowerment Phase (Weeks 1 - 12)**
2. **The Lifestyle Phase (Months 4 - 12)**

The Empowerment Phase

The goal of the Empowerment phase is to promote consistent independent physical activity and exercise. **The primary focus is on assisting older adults to achieve 150 minutes per week of moderate intensity aerobic exercise** and 2 days per week of strength training. By the end of the third month, participants should be able to maintain their new physical activity and exercise routine independently and record their exercise on the Personalized Exercise Log.

**** Note: All the participant's exercise must be recorded on the Personalized Exercise Log, regardless of whether it was completed at the YMCA during one-on-one sessions, group class, or done independently or elsewhere. This is how we will show adherence.***

Personal Coaching Sessions with participants :

Weeks 1 – 6:	2 times per week
Weeks 8, 10, 12:	1 time per week
Weeks 13-52:	1 time per month

During the Empowerment Phase, you will slowly and gradually ramp up aerobic and resistance training exercise through weekly Personal Coaching Sessions. You will empower participants to be independent with exercise by educating them on the use of aerobic and strength training machines / exercises, to the group exercise classes appropriate for each participant's fitness level and abilities. You will empower participants to achieve moderate intensity during independent aerobic workouts by helping them understand Target Heart Rate (THR) and Rating of Perceived Exertion (RPE).

You will ask participants about adverse events (AEs) at the beginning of each Coaching session and report to the LEAP! Rx study team in a timely manner. AEs are any illness, injury or medication change the participant has had since the last session.

You will provide encouragement and motivation to adhere to the program goals and hold them accountable. You will empower participants to be accountable by teaching them how to record all exercise completed, on the Personalized Exercise Log, regardless of whether the exercise is completed during a one-on-one session, group class or independently.

Empowerment Phase Personal Trainer Responsibilities:

Throughout the Empowerment Phase, during Personal Coaching Sessions, you will:

- Conduct participant orientation to the YMCA and complete 6 Minute Walk Test
- Monitor and report safety concerns (i.e. illnesses, injuries, or medication changes)
- Progress participant to safely and appropriately perform 150 minutes per week of moderate intensity aerobic exercise as primary goal
- Progress participant to safely perform 2 days per week of strength training exercise
- Teach participants how to record data on the Personalized Exercise Log
    - Hold participant accountable for exercise by reviewing the Log
    - Encourage independent aerobic and strength exercise sessions
    - Help your participant find strategies to overcome barriers and be successful
    - Identify appropriate group exercise classes and encourage participation
    - Provide personalized coaching in both aerobic and strength exercise
- Promote attendance in the monthly LEAP! Curriculum Classes
    - Assign LEAP! reading based on the Personalized Exercise Log
    - Discuss LEAP! Lifestyle Assignments and goals assigned each month

The Lifestyle Phase (Months 4 through 12)

The goal of the Lifestyle Phase is to provide encouragement and motivation to participants to continue to meet the program goals, and to attend monthly group education provided by the KU LEAP! Rx study staff.

You will meet your participants one time per month to conduct a Personal Coaching Sessions. During these sessions, you will review the participant's Personalized Exercise Log and discus areas of concern such as whether the participant is completing the exercise per protocol; logging all of the exercise, is getting bored or needs to progress or modify the current exercise program.

Lifestyle Phase Personal Trainer Responsibilities:

Throughout the Empowerment Phase, during Personal Coaching Sessions, you will:

- Monitor and report safety concerns (i.e. illnesses, injuries, or medication changes)
- Complete an Accountability Check by reviewing Personalized Exercise Log for adherence to program goals; council as needed
- Provide aerobic and/or strength exercise coaching
- Discuss personal exercise and lifestyle goals and modify as needed
- Encourage participants to attend monthly LEAP! Education Classes

Safety Monitoring

At the beginning of each Personal Coaching Session, ask your participant if they have experienced any adverse events since your last session. Adverse Events (AEs) include any illnesses, injuries or changes in their medication. If the participant reports an AE, collect as much information as you can and report it to the LEAP! Rx Study Coordinator as soon as possible. Please report all AEs regardless of whether it seems to be related to the exercise.

Things to watch for or be aware of during Personal Coaching sessions:

- Foot dragging, shuffling or stumbling on the treadmill as participants fatigue
- Changes in demeanor or alertness that may increase the risk of falling
- Ask participants to rest if they look overly fatigued or uncomfortable
- Signs of heart attack, or stroke
- If the participant is diabetic, watch for signs of hypoglycemia

LEAP! Education Classes

In addition to the monthly one-on-one Personal Coaching Sessions, participants will attend a LEAP! Education group class each month, which is based on the LEAP! Curriculum. The LEAP! Curriculum was designed by professionals at the KU ADC. This evidence-based curriculum provides practical everyday strategies to promote overall wellness and reduce Alzheimer's disease (AD) risk factors.

The LEAP! Curriculum consists of:

- The LEAP!: Brain Power Blueprint: Topics covered are:
    - Nutrition (3 classes):
        1. Healthy Fats
        2. Nutrient Density
        3. Low-Glycemic eating
    - Physical Activity and Sedentary Behavior
    - Social and Cognitive Engagement
    - Stress Management
    - Exercise
    - Sleep

LEAP! Rx Exercise Program

Aerobic Exercise

You will slowly and gradually increase your participant's aerobic exercise duration and intensity/effort (RPE) during the Empowerment Phase. Participants will reach 150 minutes of aerobic exercise by Week 6. They will reach a moderate intensity level by Week 10 that will be continued for the duration of the program. A Personalized Exercise Log outlines the progression for

aerobic exercise including duration and intensity levels. Group exercise classes are encouraged as a strategy to meet the goals, but does not substitute for one-on-one sessions.

Frequency of Exercise Sessions: As the duration and intensity increase, you will need to assist your participant in determining appropriate frequency of aerobic exercise sessions. Your participant's tolerance for the aerobic exercise will drive the duration and frequency of aerobic exercise sessions. Participants are expected to perform 150 minutes of aerobic exercise (not counting warm-up or cool-down) over 3-5 days. Their exercise tolerance will help you determine frequency of sessions. Those capable of longer duration per session, can do fewer sessions per week. Participants can complete aerobic exercise in as little as 10-minute bouts and not recommended to exceed 60 minutes in one session.

150 minutes of aerobic exercise can be broken down in the following ways:

3 days for 50 minutes + warm/cool (60 min. on Treadmill)
4 days for 38 minutes + warm / cool (48 min. on Treadmill)
5 days for 30 minutes + warm / cool (40 min. on Treadmill)

Intensity of Aerobic Exercise: Achieving and sustaining 150 minutes of moderate intensity exercise is the primary goal of the program. Thus, Personal Coaching Sessions will include teaching participants how to monitor their intensity using the Rate of Perceived Exertion (RPE) using the modified Borg scale (1-10), with a goal of working in the "somewhat hard" or "hard" range (4-5).

Rating of Perceived Exertion (RPE Scale)	
10	Maximal
9	Really, Really, Hard
8	Really Hard
7	
6	Hard
5	Challenging
4	Moderate
3	Easy
2	Really Easy
1	Rest

LEAP! Rx
LIFESTYLE EMPOWERMENT FOR ALZHEIMER'S PREVENTION

Aerobic Exercise modality *(type)*
We recommend that participants walk on the treadmill as their primary exercise modality to best influence their cardiorespiratory fitness. Use of other aerobic modalities is allowed, though not preferred. Use your professional judgement on whether the participant should be using a different modality due to issues such as balance, boredom, or overuse injury from the treadmill walking (e.g., shin splints, foot, knee or hip pain, etc.).

Make it clear to the participant that if they decide they would like to try a different aerobic machine, such as a stationary bike or elliptical, to please request this during one of their coaching sessions. This will allow you to ensure their safety and assist in selection of appropriate settings on the

machine. Ask them not to switch to another aerobic modality during independent exercise without instruction from you.

Strength Exercise

Current guidelines for strength training include a minimum of 1 set of 8 – 12 repetitions for 8 – 10 exercises covering the major muscle groups. Help the participant choose exercises they can perform safely on their own, with consideration of common issues among older adults, such as balance and musculoskeletal issues. You have flexibility in designing a strength training program with your participant. You can use machines, free weights, balls, bands, tubing, etc. Keep in mind when developing strength exercise programs for the participant, most of their strength exercises will be completed independently, therefore safety is the primary concern. Group exercise classes are encouraged as a strategy to meet goals, but does not substitute for one-on-one sessions.

Independent Exercise

To meet the program goals, participants will be expected to perform independent exercise sessions for both aerobic and strength, beginning the first week of the program. Encourage participants to fully utilize their YMCA membership and perform their exercise at the YMCA. If the participant wants to exercise at home or otherwise outside of the YMCA, it is permissible, but not preferred.

All exercise sessions – at the YMCA, home, or while traveling – will be recorded in the Personalized Exercise Log. The Personalized Exercise Log will stay at the YMCA – participants will not take the log home. They will need to record all of their exercise on the Personalized Exercise Log independently. Therefore, it will be important you assure they know how to log all of their exercise. Remember to ask your participant each time you meet with them if they have completed independent exercise sessions and whether they recorded it appropriately.

Group Exercise

As the LEAP! Rx Coach, you will encourage participants to attend group fitness classes as a strategy to achieve or exceed the aerobic and strength exercise goals. These sessions will not replace the Personal Coaching Sessions.

Using the YMCA Group Class schedule for the specific location, LEAP! Rx Coaches will identify 2 - 3 LEAP! Rx-approved group exercise classes appropriate to the participant's fitness level. Beginner level classes will be recommended during the Empowerment Phase (Months 1 – 3); Intermediate classes during the early months of the Lifestyle Phase (Months 3 – 6) and advanced classes during Months 6 – 12.

Group exercise classes should be > 30 minutes in duration with an expected RPE of 3 to 5 to be sufficient to achieve minutes towards either the aerobic or strength training exercise.

Examples of Beginner/Intermediate Level Group Exercise Classes:

- Silver Sneakers Classic
- Silver Sneakers Circuit
- AOA Resistance
- Walk, Talk and Tone
- AOA Low Impact
- Zumba Gold
- Water Fitness

- Deep Water Fitness

Examples of Intermediate/Advanced Level Group Exercise Classes:

- Resistance Training
- Strength Training
- LesMills BODYPUMP
- Zumba
- Cycling

Completing the Personalized Exercise Log

The LEAP! Rx Coach is responsible for assuring the Personalized Exercise Log is filled out completely and accurately. The information recorded on the Personalized Exercise Log is important for monitoring adherence. Additionally, some of this information will be shared with the participant's referring physician. You will need to take plenty of time to assure participants know how to record their exercise. You will record it for them during the first exercise session and then have them start recording it at following sessions to promote independence. All exercise completed by the participant must be reflected on the Personalized Exercise Log regardless of location of exercise (YMCA, home or other).

The duration of exercise will be entered in minutes, excluding time spent warming up and cooling down. Their effort (RPE) level will be rated and recorded using the modified Borg scale (1-10). Additionally, indicate whether the session was one-on-one with the LEAP! Rx Coach, independent, or in a group class.

Group exercise should be logged appropriately as minutes of aerobic or minutes of strength exercise. Teach participants difference between the two, and to take a mental note of how much time was spent during the class, doing aerobic or strength exercise.

Information included on the Personalized Exercise Log:

- The week's goals for exercise, including duration, frequency, intensity
- Weekly highlights and/or changes, such as an increase in intensity or duration
- Monthly group LEAP! Education reading assignments
- A place to write notes and a reminder to ask about adverse events

Accountability and Compliance

Accountability check

As a LEAP! Rx Coach, it is important to set clear goals and hold the participants accountable for meeting the exercise goals and attending group exercise sessions and monthly LEAP! Education classes. It is your responsibility to monitor attendance, compliance and hold the participant accountable.

During each Personal Coaching Session you will:

Conduct an accountability check:

- Review the Personalized Exercise Log with the participant to determine whether they are meeting the goals of the program
- Review and encourage opportunities to participate in group exercise
- Discuss barriers to meeting the exercise goals and strategies to overcome them
- Modify aerobic and strength exercises as needed

- Praise participant for their accomplishments
- Provide encouragement to actively engage in the LEAP! Rx Program

Missed exercise (vacation, illness and injury)

It is expected that participants will miss periods of exercise due to illness, vacation or other reasons. When a participant misses two or more consecutive weeks of exercise, the LEAP! Rx Coach should adjust exercise parameters to work up to their prior intensity and duration before they stopped exercising. To a degree, this can be individualized by the LEAP! Rx Coach following the general guideline that exercise parameters should be reduced for each consecutive week missed.

For example, if the participant:

- Completed exercise through week 16, then missed 4 weeks due to vacation:
  - Week 16 – 4 weeks = Resume exercise at Week 12 intensity
- Continued to exercise while they are on vacation or otherwise outside the YMCA:
  - No modification is necessary
- Stopped exercising due to illness or injury:
  - Further modification may be required

No-shows and non-compliance

Participant attendance is vital to meet the goals of the program. As a LEAP! Rx Coach, it is your responsibility to monitor attendance, compliance, and hold the participant accountable.

If a participant fails to show up for an appointment:

- Call participant within 15 minutes of scheduled start time
  - Ask participant if they are ok and discuss reason(s) for the missed exercise session; reschedule exercise session.
  - If they are missing sessions due to an AE, gather as much information as you can, and report it to the LEAP! Rx Study Coordinator as soon as possible.
- If they do not answer, leave a message and call the participant the following day to reschedule
- If you are unsuccessful after several days, notify LEAP! Rx Study Coordinator via secure email or phone call.

If a participant is not achieving their goals:

Address their individual exercise goals during a Personal Coaching session, discuss barriers and find strategies to overcome the barriers. If boredom is an issue, change it up. Offer to teach the participant a different aerobic modality such as an elliptical or stationary bike. Provide new strength training exercises, encourage group exercise for the social interaction, etc. Don't forget to ask if they have experienced injury or illness since you last saw them and adjust exercise accordingly.

LEAP! Rx

LIFESTYLE EMPOWERMENT FOR ALZHEIMER'S PREVENTION

Participant Orientation

KU ALZHEIMER'S DISEASE CENTER

The University of Kansas Medical Center

Participant Orientation will be conducted at the assigned exercise facility, in 2 parts: 1) by LEAP! Rx study staff and 2) the assigned YMCA personal trainer at the facility.

The purpose of the orientation is to assure the participant knows exactly what is expected of them for the study and for the 1-year exercise and education program.

Upon completion of Orientation, the YMCA LEAP! Rx Coach will introduce the strength training, conduct a 6 Minute Walk Test and an aerobic exercise session with the participant. During the Aerobic exercise session, trainer will introduce Target Heart Rate and Rating of Perceived Exertion. Following the aerobic exercise session will teach the participant how to log their minutes in the Personalized Exercise Log.

Other?

LEAP! Rx Study Staff (KU) Conducts the following:

Review Study's Purpose
Review Program Components
Review Program goals and the Personalized Exercise Log
Getting credit for and logging your exercise minutes
Reporting illnesses, injuries, and medication changes
Attending Monthly LEAP! Rx Curriculum (Group Education Classes)

LEAP! Rx Coach (YMCA) Conducts the following:

Provide Facility tour and build rapport
Introduce Strength Training Area
Discuss Interests, barriers and strategies for meeting program goals
Complete an Adverse Event Check
Complete an Aerobic exercise session
Introduce Target Heart Rate and Rating of Perceived Exertion

LEAP! Rx Study Staff (KU) Conducts the following:

____ **Welcome Packet and Review Study's Purpose**

The purpose of the study is to learn whether exercise and education on healthy lifestyle choices can help older adults meet current physical activity guidelines, improve fitness, and result in improvement in other health measures, such as cholesterol and blood sugar.

____**Define Program Components**

The current guidelines for physical activity addressed in the program are:

- **150 minutes of moderate intensity aerobic exercise per week.** Aerobic exercise is any exercise that is sustained for several minutes (without rest) that increases your heart rate and makes you breathe harder. Walking is a common aerobic exercise.
- **2 days per week of strength training (i.e., lifting weights).** Strength training consists of moving your body and limbs against a resistance, typically with rest breaks. Lifting weights is a common strength training technique.
- **One-on-One Personal Coaching Sessions** with a certified personal trainer who will teach you fun and effective strategies to meet the current exercise guidelines for older adults.
- **Group exercise classes** as an option for meeting your weekly exercise goals.
- **Garmin VivoFit3 activity tracker** is provided as a motivational tool to encourage you to increase your daily steps as you ramp up your exercise and physical activity.
- **Monthly Group Education using the LEAP! Curriculum** to learn healthy lifestyle behaviors to reduce your risk for Alzheimer's disease.

____ **Discuss Program goals and Introduce the Personalized Exercise Log**

- Finding weekly goals and determining when weekly goals change
- Primary focus on 150 minutes/week of moderate intensity aerobic exercise
    - Starts at 60 minutes, gradually increases to 150 minutes over 6 weeks
    - Gradual increase in intensity/effort (THR & RPE) over 9 weeks
- Engage in 2 days per week of resistance training
- Meet with your LEAP! Rx Coach for one-on-one exercise sessions
    - 2x/week for the first 6 weeks
    - 1x/week during Weeks 8, 10, 12
    - 1x/month during Weeks 13 – 52
- Schedule your sessions directly with your trainer
    - Choose consistent days/times when possible
    - Inform your trainer well in advance when you need to reschedule
    - Inform your trainer of all travel plans to devise an exercise plan
- Exercise Independently starting in Week 1
    - Complete independent sessions at the Y when possible

    - Incorporate group exercise classes as a strategy to reach goals
    - Prevent injury by learning, from your trainer, how to use a variety of aerobic exercise machines.
    - Learn safe and appropriate techniques and strategies for independent exercise during one-on-one trainer sessions.
    - If you make a mistake, draw a single line through the error, write the correct data and initial and date. (Example: ~~10~~ 15 2/4/19 ABC)
      Please do not use white out!
- Wear your Garmin VivoFit3 and increase your daily steps over time.

____ **Getting credit for your exercise**

- Remember, information about your participation in the study is being shared with the physician who referred you to the Program, so you will want to get credit for all of your hard work.
- Learn to be independent with recording your exercise minutes on the Personalized Exercise Log, so you can log all of you exercise minutes on your own. Remember, this information is being sent to the physician who referred you to the Program.
- Warm up and cool down minutes are not counted toward exercise minutes
- Every entry should have duration, effort (RPE 1 – 10) and supervision type (i.e. One-on-one, group class or independent)
- Logging group classes can be tricky, work with your trainer to determine whether a class is aerobic (cardio) or strength (resistance) or both. Record the duration accordingly.

____ **Reporting illnesses, injuries, and medication changes**

- We want you to report all illnesses, injuries, or medication changes, regardless of whether you feel they are related to the study or the exercise.
- Trainers will ask you about changes to your health at every one-on-one session; study staff may call to follow up
- Study staff will call you periodically to check as well
- Depending on the AE, changes to your exercise program may be necessary and possibly your physician's clearance to continue.

____ **Attend Monthly LEAP! Rx Curriculum (Group Education Classes)**

- Monthly Topics:
- Nutrition: 1. Healthy Fats; 2. Nutrient Density; 3. Low-Glycemic eating
- Physical Activity and Sedentary Behavior
- Social and Cognitive Engagement
- Stress Management
- Exercise
- Sleep

LEAP! Rx Coach (YMCA) Conducts the following:

____ **Provide Facility tour and build rapport**

- Provide a tour and overview of YMCA, benefits of membership, and check-in process
- Review what to wear and bring to each session
- Provide group exercise class schedule and encourage them to try a class this week and briefly discuss their experience at Session 2 this week (or the next scheduled session)

____ **Introduction to Strength Training Area**

- Briefly provide your participant a tour of the strength training area, noting machines that are for upper body exercise and those that are for lower body exercise. Discuss with your participant any musculoskeletal issues, muscle imbalances, past injury, etc. to further assist you in developing a strength training plan appropriate for the participant. Week 1 Session 2 will be ½ aerobic exercise and ½ strength training.

____ **Discuss Interests, barriers and strategies for LEAP! Rx Coaching Sessions**

- Past exercise experiences, motives/incentives, intended outcomes, interests
- Factors / barriers that may impact successful study participation and completion

____ **Complete an Adverse Event Check (AE) and AE form in REDCap**

- Prior to starting each Personal Coaching Session (including the 6MWT test), determine whether it is safe for the participant to exercise. Check to see if they had an Adverse Event (AE), i.e., illness, injury, or medication change.
    - If an AE occurred and it is not safe to perform the 6MWT, do not proceed. Collect as much information about the AE as you can and inform the LEAP! Rx Study Coordinator following this session.
    - If an AE occurred, but it is still safe to perform the 6MWT, collect as much necessary information about the AE as you can, then proceed with the test, and complete report to the LEAP! Rx Study Coordinator following this session
    - If an AE did not occur, proceed with the 6MWT no other reporting necessary.

_____ **6-Minute Walk Test (15 minutes)**

Complete this prior to aerobic exercise. Instructions and the data collection sheet are included in the Personalized Exercise Log.

____ **Complete aerobic exercise session, instill Independence, introduce Target Heart Rate and Rating of Perceived Exertion (35 minutes)**

- Demonstrate all safety features, starting and stopping, increasing the speed and incline, and using the heart rate sensors.

- Have the participant do a 5-minute warm up with the intention of reaching the lower end of the THR zone by end of the 5 minutes, walk for 20 minutes in the THR zone provided, cool down for about 5 minutes.
- Have the participant increase the speed or incline as needed and to use HR sensors to promote independence.
- Discuss the relationship between heart rate and rating of perceived exertion so the participant can appropriate judge their intensity during independent exercise sessions
- Show the participant appropriate stretches to perform after each treadmill session.

____ **Record data on the Personalized Exercise Log**

- Teach your participant to be independent with logging their exercise.
- Train them to record duration in minutes and to record their effort or rating of perceived exertion.
- Warm up and cool down minutes do not count toward their exercise minutes
- Teach the participant that every entry should have a duration in minutes, effort from 1 – 10, and supervision type as "One-on-One", "Group Class", or "Independent"

____ **Review group exercise class schedule (10 minutes)**

- Strongly encourage participants to attend group classes, starting this week, as a strategy to achieve the exercise goals for both aerobic fitness and strength.
- Warm up / cool down components are not counted as exercise minutes
- Aerobic and strength components of the class are recorded on the Personalized Exercise Log accordingly. Train participants to keep a mental (or written) note as to the duration of aerobic and strength exercise components and you will help them record on the Personalized Exercise Log during your next session.
- Review the group exercise class schedule and answer questions. Identify and highlight or mark 2-3 classes that have both aerobic and strength components and are appropriate for the participant.

____ **Session wrap-up and one-on-one trainer schedule**

- Encourage and remind participant to perform 1 independent aerobic exercise session this week, or do group exercise if they prefer or are not ready to be on the their own.
- Schedule 1 additional training session for this week
- Based on the supervised session schedule below, schedule future sessions. Be consistent with days and times, when possible:

  2x/week for the first 6 weeks
  1x/week during Weeks 8, 10, 12
  1x/month during Weeks 13 – 52

Participant Orientation

LEAP! Rx
LIFESTYLE EMPOWERMENT FOR ALZHEIMER'S PREVENTION

Participant Expectations

Example Exercise Logs

KU ALZHEIMER'S DISEASE CENTER
The University of Kansas Medical Center
