# Supplementary material for: Lifestyle empowerment for Alzheimer’s prevention prescribed by physicians: Methods and adaptations to COVID-19
Source: Contemp Clin Trials. Author manuscript; Available in PMC 2025 Mar 24. (PMC11932157; doi:10.1016/j.cct.2024.107729)
Supplement: Participant Expectations [file NIHMS2037346-supplement-Participant_Expectations.pdf]

LEAP! Rx

LIFESTYLE EMPOWERMENT FOR ALZHEIMER'S PREVENTION

Participant Expectations

KU

ALZHEIMER'S DISEASE CENTER

The University of Kansas Medical Center

LEAP! Rx Program Overview & Goals

LEAP! Rx
LIFESTYLE EMPOWERMENT FOR ALZHEIMER'S PREVENTION

GOAL #1 Meet current exercise guidelines to improve fitness:
150 min./wk. aerobic — 2x/wk. strength

GOAL #2 Learn about healthy lifestyle behaviors to reduce your risk for Alzheimer's disease

PHASE 1: The Empowerment Phase (Weeks 1-6)

GOALS

- **#1:** Learn effective techniques for aerobic & strength exercise
- **#2:** Gradually work up to 150 min./week of aerobic exercise
- **#3:** Gradually work up to 2 days/week of strength training
- **#4:** Develop the independence to do #2 & #3 on your own

Empowerment Phase Timeline:

Weeks 1-6	Coaching 2x/week	Exercise independently: Meet remaining goals	Group exercise optional
	Wear a Garmin Vivofit3 activity tracker to help monitor your physical activity.		

Log all exercise

Questions? Ask your Coach.

PHASE 2: The Lifestyle Phase (Months 2-12)

GOALS

- **#1:** Keep up the good work!
- **#2:** Learn even more fun & effective exercises with your coach.
- **#3:** Attend a monthly group education session to learn healthy lifestyle strategies

Lifestyle Phase Timeline:

MONTHS 2-12	Coaching 1x/month	Exercise independently: 150 min. aerobic & 2x strength per week	Group ex. optional	Lifestyle class 1x/mo.
	Wear a Garmin Vivofit3 activity tracker to help monitor your physical activity.			

Log all exercise

Questions? Ask your Coach.

LEAP! Rx Study Expectations

LEAP! LIFESTYLE EMPOWERMENT FOR ALZHEIMER'S PREVENTION Rx

Welcome to the LEAP! Rx Program. We are so excited you joined the Program!

Define Program Components

150 minutes of moderate intensity aerobic exercise per week. Aerobic exercise is any exercise that is sustained for several minutes (without rest) that increases your heart rate and makes you breathe harder. Walking is a common aerobic exercise.

2 days per week of strength training (i.e., lifting weights). Strength training consists of moving your body and limbs against a resistance, typically with rest breaks. Lifting weights is a common strength training technique.

Group exercise classes are an option for meeting your weekly exercise goals.

Garmin VivoFit3 activity tracker is provided as a motivational tool to encourage you to increase your daily steps as you ramp up your exercise and physical activity.

Group Education Curriculum is found in the LEAP! Brainpower Blueprint Book.

One-on-one training sessions with LEAP! Rx Coach

Meet Program Goals

Where to find weekly goals

- Aerobic and resistance exercise goals can be found at the top of your personalized exercise log
- If a goal changes from the previous week, it is circled

Primarily focus on meeting 150 minutes/week of aerobic exercise at the proper intensity

- **Weeks 1–6:** Gradual Increases in Exercise Duration
- **Weeks 1 – 9:** Gradual increases in Exercise Intensity
- **Weeks 10 – 52:** Exercise Duration and Intensity Constant

Meet with your LEAP! Rx Coach for one-on-one exercise sessions

- 2x/week for the first 6 weeks
- 1x/week during Weeks 8, 10, 12
- 1x/month during Weeks 13 – 52

Schedule your sessions directly with your trainer

- Choose consistent days/times when possible
- Inform your trainer well in advance when you need to reschedule
- Inform your trainer of all travel plans

LEAP! Rx Study Expectations

Meet Program Goals (Continued)

Exercise independently starting week 1

Make a plan with your trainer of how to be independent and meet your goals

Complete independent exercise at the Y when possible

Keys to success to meet goals

Prevent injury by learning to exercise using various pieces of aerobic equipment. Learn how to use new equipment during one-on-one sessions to assure your safety.

Have fun, add variety, and make friends while meeting your goals.

Learn about group exercise classes, which classes are appropriate for you, and how they apply to meeting your goals

Meet the right intensity by learning to use the heart rate sensors on the machines to achieve target heart rate goals and by learning to gauge your Rating of Perceived Exertion (RPE).

How to get credit for exercise

Record your exercise in the Personalized Exercise Log

Be independent with logging your exercise

Make sure that every exercise session is logged. If you are doing the exercise away from the gym, be sure to record it.

Warm up and cool down minutes are not counted towards exercise minutes

Every entry should have a duration in number of minutes, effort from 1-10, and supervision of either "One-on-one", "Group Class", or "Independent"

If you make a mistake, draw a single line through the error, write the correct data and initial and date. (Example: ~~10~~ 15 2/4/19 ABC)

Illnesses, injuries, and medication changes (Adverse Events)

Report Adverse Events to your trainer and / or study staff

Report all AEs whether exercise related or not

Study staff may call you to follow up and gather more information

Depending on the AE, changes to your exercise program may be necessary and possibly your physician's clearance to continue

Brain Health Boot Camp

Week	Topic
1	Understanding Alzheimer's Disease + Physical Activity
2	Exercise
3	Getting Started with a Brain Healthy Diet
4	Nutrient Density, Low-Glycemic Eating, Healthy Fats
5	Social & Cognitive Engagement
6	Sleep Health & Stress Management

The Brain Health Boot Camp is a 6 week course covering each of the above topics. Classes are live through Zoom on Mondays at 1pm for 6 straight weeks. The next course begins February 13th, 2023. The study team will follow up through email with a link for class sign up if interested.
