# Supplementary material for: Lifestyle empowerment for Alzheimer’s prevention prescribed by physicians: Methods and adaptations to COVID-19
Source: Contemp Clin Trials. Author manuscript; Available in PMC 2025 Mar 24. (PMC11932157; doi:10.1016/j.cct.2024.107729)
Supplement: Supplemental Material [file NIHMS2037346-supplement-Supplemental_Material.docx]

**Supplemental Material 1.**

2.4.1 LEAP! Rx Coach

Each fitness center will designate a LEAP!Rx Coach. The program design is based on the Behavioral Choice Theory. [64] LEAP! Rx Coaches will use motivational interviewing to assess readiness for change, motivations, goals, and barriers to physical activity. LEAP! Rx Coaches will have the following responsibilities:

- Function as the primary point of contact for study staff and participants
- Set individualized goals, monitor m-Health physical activity levels, review progress, and provide counseling on exercise, nutrition, and body weight.
- Lead exercise training sessions for supervision and training in resistance and aerobic exercise
- Attend monthly study meetings with area LEAP! Rx Coaches and study staff

2.4.2 LEAP! Coach Training and Qualifications

LEAP!Rx Coaches will have a personal trainer or fitness instructor certification from a nationally recognized organization. In addition, LEAP! Rx Coaches are required to complete the four-phase training of the ACE Integrated Fitness Training Model certification. This ACE certification prepares Coaches to deliver custom, individualized exercise programs with goals ranging from simple function to health, fitness, and elite performance. The training manual provided to trainers is included in Supplemental Material 2.

2.4.3 Training Fidelity Plan

To enhance the fidelity of the delivery of the LEAP! Rx Program across fitness centers and to prepare LEAP! Rx Coaches for the broad health issues of an older population:

- LEAP! Rx Coaches will undergo a training program conducted by study personnel. The basis of this training will include a manual of procedures (MOP) modified from the YMCA's recently developed "Coaching Connection" manual. This includes training on theories of behavior change and techniques for motivational interviewing. In the event of staff turnover, new LEAP! Rx Coaches are trained individually by study personnel.
- LEAP! Rx Coaches are trained in the exercise protocol and receive a manual developed over our several previous YMCA-based exercise trials. The manual will outline proper procedures for safe aerobic and resistance training progression and instruct on data collection and adverse event reporting.
- Ongoing training will be supported by close communication between LEAP! Rx Coaches and study staff. The LEAP! Rx study coordinator will lead monthly meetings bringing together the LEAP! Rx Coaches from the YMCA centers will discuss the program with study staff and troubleshoot any problems.
- We will intermittently (monthly) monitor coaches for protocol compliance and professionalism. Booster training sessions will be developed and employed as needed.

A copy of the Leap! Rx trainer certification checklist is included in Supplemental Material 2.

2.4.4 Exercise Training Program

The LEAP! Rx exercise program consists of an intensive 12-week Empowerment Phase followed by a 40-week Lifestyle Phase (See Supplemental Table 1.). The overall goal of the training is to meet current recommendations of 150 minutes a week of aerobic exercise (over 3 to 5 days a week) and 2 days a week of resistance exercise.[33, 34] Trainers and participants can work together to create an exercise program that best suits the participants’ needs and physical abilities. This flexibility represents our efforts towards implementation science, translating restrictive laboratory findings into “real-world” settings. This facilitates independence and self-reliance; personal training supervision will decrease frequency throughout the 12-week Empowerment phase, followed by monthly sessions during the Lifestyle phase. Participants are expected to exercise independently (home or at the YMCA) to meet the overall goal. The gradual introduction and progressively increasing volume and intensity of exercise are designed to build self-reliance.

LEAP! Rx Coaches will supervise in-person exercise training sessions. Coaches will also work with participants to set individualized goals and monitor progress toward those goals. One-on-one coaching will occur as part of the exercise training during the Empowerment phase and monthly afterward. Coaches will work to identify barriers and provide guidance for achieving the participant's physical activity, fitness, nutrition, and other LEAP! Rx goals. LEAP! Rx Coaches will encourage participation in monthly educational sessions and group exercise opportunities.

Individuals may need to exercise from home with limited monitoring in an emergent situation. Study staff will provide publicly or privately available exercise videos online and instructive handouts and will make as many check-in calls as possible. Videos are scientifically vetted or come from highly reputable sources (e.g. AHA or ACSM) and will attempt to support the existing protocol as much as possible. Online platforms are used to communicate with participants using various means, including email and phone, as well as standard computer and phone apps that could provide a platform for face-to-face communication. These apps may include FaceTime, Skype, Zoom, or Microsoft Teams or similar apps. If these are used, we will do so on consultation with investigators who have ongoing telehealth research and apply their standard procedures for privacy. Handouts will encourage exercises that are consistent with public health recommendations and publications. Check-in call frequency will vary based on the situation, but we will target a biweekly call.

2.4.5 Empowerment Phase

The Empowerment phase comprises 12 weeks of supervised and unsupervised exercise training sessions. Aerobic exercise duration will be gradually increased over six weeks to a goal of 150 minutes per week. Exercise will be primarily supervised in the Empowerment phase and follow our protocol used in NIH-funded trials [8, 65] to successfully introduce and increase the volume of exercise in older adults. Participants will perform 60 total minutes of aerobic exercise in the first week and increase that volume by about 18 minutes per week to achieve 150 minutes per week by 6 weeks. Coaches will supervise during this titration period two times per week from Weeks 1-6 and one time per week in Weeks 8, 10 & 12. By Week 6, participants will walk, cycle, or elliptical for 150 minutes a week over 4-5 days at a level deemed "somewhat hard" or 3 to 5 (out of 10) on the modified Borg Rating of Perceived Exertion scale (RPE). [66] The use of an exertion rating as a proxy for a target heart rate zone decreases complexity allows greater individualization in the context of bradycardic drugs and is a validated method of exercise prescription. During supervised sessions, participants are taught how to rate their exertion levels based on their heart rate.

Participants will also be encouraged to perform resistance exercises twice a week. Resistance exercise will target the major muscle groups (e.g., pectorals, rhomboids, and latissimus dorsi, biceps, triceps, quadriceps, hamstrings, calves). During Weeks 1-6, LEAP! Rx Coaches will work with participants to identify exercises and machines that are comfortable for the participant. Resistance will be set using the RPE to a resistance that is "somewhat hard" (RPE 3-5) to lift ten times. RPE is a valid method for gauging resistance training intensity like aerobic training.[67] Coaches will counsel participants on modifying exercises and resistance as participants increase strength.

Participants are encouraged to attend group exercise classes to meet the 150 minutes per week of aerobic exercise and two days per week of resistance training. Silver Sneakers classes are promoted as a good choice for participants. The details regarding the intervention are provided in Supplemental Table 2.[68]

2.4.6 Lifestyle Phase

The Lifestyle phase will consist of 40 weeks of once-monthly (every four weeks) exercise training sessions. Contact with the LEAP! Rx Coach will encourage adherence to the exercise program, assure the progression of exercise routines, and allow continual review of progress toward goals and monitoring of the m-Health data. These one-on-one training sessions will include continued goal-setting sessions to review goals, progress towards goals, and discuss other lifestyle factors such as nutrition, body weight, smoking cessation, etc. The goal of the Lifestyle phase is to continue to safely challenge the participant whiRx le allowing greater independence in the timing and location of exercise.

2.4.7 Mobile-Health (m-Health) Monitoring

The LEAP! Rx Program will leverage emerging m-Health technology to objectively measure physical activity and provide this data to the participant (self-monitoring), LEAP! Coaches, and ultimately the referring clinician (i.e., physical activity as a vital sign). Each participant will be given an m-Health monitoring device worn on the wrist (Garmin Vivofit2, Garmin, Olathe, KS). Devices store up to 30 days of data and have a battery life of 1 year. Data from these devices are collected through smartphone, computer or wireless access points (Garmin Vivohub2) that are installed at each of the YMCA sites and the participants smartphone (if available). Simply walking by a wireless access point will convey this data to our centralized database; thus, personal smartphones and computers are not required. LEAP! Rx Coaches will access an online customized dashboard (MyInertia) that provides them with access to their participants' data. LEAP! Rx Coaches will either print a monthly hard copy or electronically view the physical activity report for their participants so that smartphones and computers are not required to access their data. At the end of study participation, LEAP! Rx Coaches will train the participants, whenever possible, to access their data through a device (i.e., smartphone or tablet) or on their home computer so they can continue using the devices.

2.4.8 Group Exercise Sessions

Each YMCA has a variety of group exercise opportunities available to YMCA members. Participants are encouraged to attend group exercise classes to augment their individualized, supervised exercise training sessions or home-based independent exercise. Group exercise opportunities are categorized into Beginner, Intermediate, and Advanced classes so that participants can appropriately progress their exercise volume and intensity. Beginner-level courses are encouraged during the initiation phase, Intermediate during weeks 12 to 24, and Advanced during weeks 25 to 52. LEAP! Rx Coaches will assist in identifying classes that will effectively fulfill aerobic and resistance exercise goals.

2.4.9 Smart Aging Educational Curriculum

The Smart Aging Educational Curriculum will be delivered in person at the KU Clinical Research Center by KU ADRC study staff with an alternate option for viewing online. The overall goal of the curriculum will be to initiate and sustain behavior change and provide a framework for lifestyle enhancement. Although each session will include some didactic information, the sessions will primarily focus on group discussion and goal-setting. The Smart Aging Curriculum has been developed and deployed at the KU ADRC. We will use a local media company to create and translate the existing KU ADRC programming into consumer-friendly and professionally packaged materials. To support the delivery of the program, YMCA staff will receive training materials. Sessions will include online videos guided completion of a workbook with a LEAP! Rx Coach that: 1) asks participants to reflect on their habits indicated by monitoring and changes with age, 2) sets realistic and measurable goals, describes expectations for change in behaviors and barriers to making change, 3) evaluates perceived benefits of increasing activity, reinforces strategies for rewarding behavior change and enjoyable activities to pursue, ratings of confidence in the ability to make changes, 4) tips for ways to change behavior (generic and targeted to individual habits/barriers). The educational and goal-setting piece is based on Social Cognitive Theory and Behavioral Choice Theory concepts. [64]

Supplemental Table 1. LEAP! Rx Program Events

| **Initiation Phase** | | | | **Maintenance Phase** |
| --- | --- | --- | --- | --- |
|  | **Weeks**  **1-4** | **Weeks**  **5-8** | **Weeks**  **8, 10, 12** | **Weeks 13 to 52** |
| **Supervised Exercise Training** | 3 per week | 2 per week | 1 per week | Every 4 weeks (10 sessions) |
| **Group Exercise** | Weekly | Weekly | Weekly | Weekly (40 sessions) |
| **Smart Aging Curriculum Class** | Monthly | Monthly | Monthly | Every 4 weeks (9 sessions) |

Supplemental Table 2. Descriptors for exercise training intervention

| **Descriptor** | **Training Recommendation** |
| --- | --- |
| Frequency |  |
| *Aerobic exercise* | 3-5 days per week |
| *Strength training* | 2 times per week |
| *Felxibility training* | 2 times per week |
| Intensity |  |
| *Aerobic exercise* | Moderate to Vigorous Intensity, RPE 4-5 |
| *Strength training* | RPE 4-5 |
| Time |  |
| *Aerobic exercise* | 150 minutes per week |
| *Strength training* | Minimum of 1 set of 8 – 12 repetitions for 8 – 10 exercises covering the major muscle groups, exercises determined by participant and Leap! Rx coach |
| Type |  |
| *Aerobic exercise* | Walking on treadmill recommended, but different aerobic machines such as stationary bike or elliptical may be selected |
| *Strength training* | Machines, free weights, balls, bands, tubing, etc. Group classes may also be selected. |

**Supplemental Material 2.**

**Supplemental Material 3.**

2.8.1 Additional Power Details

We anticipate the effect size (mean difference divided by the standard deviation) to be half the effect of that observed in our 6-month trial[31] the exercise intensity will likely be lower than observed in a tightly controlled research protocol. Additionally, we anticipate a 20% decrease in this effect size at the 52-week endpoint compared to the 12-week effect. We assume that the 12- and 52-week time point results will be positively correlated; thus, the probability of rejecting the null hypothesis of no group difference at both 12 weeks ($\mathrm{Re}j_{12 wks}$) and 52 weeks ($\mathrm{Re}j_{52 wks}$) is $\Pr\left( \mathrm{Re}j_{12 wks}\cap Rej_{52 wks} \right)=\Pr\left( \mathrm{Re}j_{12 wks} | \mathrm{Re}j_{52 wks} \right)Pr(Rej_{52 wks})$

We use this form of joint distribution because we anticipate the effect size to be lower for the 52-week time point; hence, our power estimates are conservative. To estimate the joint probability of rejecting both, we anticipate that if the effect is sustained (i.e., $Pr[Rej_{52 wks}]$, Then, the intervention group will likely have increased VO_2peak_ during the 12 weeks. Thus, under the condition$\mathrm{Re}j_{52 wks}$, the conditional probability of $\mathrm{Re}j_{12 wks}$, or $Pr(Rej_{12 wks}|Rej_{52 wks})$ should be high (~1.0). Therefore, $\Pr\left( \mathrm{Re}j_{12 wks} | \mathrm{Re}j_{52 wks} \right)Pr(Rej_{52 wks})\approx Pr(Rej_{52 wks})$. Using this, we can approximate the power based on data for a single time point.

Our prior study observed an effect size of 1.2 in the exercise intervention groups.[31] We conservatively assume the intervention effect will be approximately half that (0.6). As indicated above, we anticipate the 52-week time point to have a 20% decrease for the sustained effect, reducing the anticipated effect size to 0.48. Our sample of 110 subjects per group will have over 88% power while allowing for 20% attrition (leaving approximately 88 subjects per group). With even smaller effect sizes (e.g., 0.45) this sample size will still have 84% power (nQuery Advisor® 7.0, 1995-2007).

2.9.1 Aim 1

Study Aim 1 is to assess the implementation and scalability of the LEAP!Rx Program for clinicians and patients. We will test the efficacy of an exercise and healthy lifestyle program using a unique referral method embedded in the electronic health record (EHR) as compared to the standard of care.

Descriptive statistics for referral status, randomization group, retention, and demographics will be calculated. Statistical tests will be conducted to compare differences in referral status, retention, and demographics, including chi-squared tests for categorical variables and t-tests for continuous variables.

Patient-centered outcomes will also involve generalized linear mixed models (GLMMs) at baseline, 12-, and 52-week time points. For continuous measures, the LMMs (a special case of the GLMMs) will suffice. In contrast, other more discrete-measured variables will require different model assumptions (such as the multinomial distribution with an ordinal logit function, similar to that for the clinician measures), such as with Likert-type measures. Model assessments will include residual analyses (to assess mean-to-variance relationships and whether they are in keeping with the exponential family distribution selected for the particular GLMMs) and observed versus expected comparisons. We will utilize the two degree-of-freedom testing paradigms previously described to assess sustained benefit simultaneously and better control type I errors

2.9.2 Aim 2

The goal of Aim 2 is to determine the effect of the LEAP! Rx Program on cardiorespiratory fitness. We hypothesize that the LEAP! Rx Program will increase and maintain cardiorespiratory fitness (peak oxygen consumption [VO_2peak_]) at both 12 weeks (after the initiation phase) and 52 weeks (after the maintenance phase). Descriptive statistics will be generated for all measures. Continuous measures will include the mean, standard deviation, percentiles, and range; categorical measures will include frequencies and relative frequencies. Bivariate analyses comparing the two treatment groups will consist of visual inspection (e.g., box plots for continuous measures) and statistical tests such as the two-sample t-test, Pearson's chi-square test, or analogous nonparametric measures as indicated based on assessments of underlying assumptions (e.g., residual analysis for t-tests, expected cell counts for Pearson's chi-square tests).

The primary study measure, VO_2 peak_, will have repeated measures over time (baseline, week 12, and week 52). Thus, we will use linear mixed models (LMMs) for analysis. Estimated linear contrasts will assess change (baseline to 12 weeks and baseline to 52 weeks) in VO_2_ peak comparing active vs. control groups, providing a randomized, intent-to-treat comparison. Our primary research hypothesis (Aim 1) involves a two-degree-of-freedom test. To conclude with our research hypothesis of a benefit at 12 weeks sustained at 52 weeks, a contrast matrix will simultaneously test in a single statistical test that VO_2peak_ increases over baseline at 12 weeks and 52 weeks.

We will use a type I error rate of 0.05 for this simultaneous two-degree-of-freedom comparison of the primary measure (F-test). Tests of secondary measures, hypotheses, and per-protocol analyses (based on ≥80% adherence) will follow a similar LMM approach. Further, we will collect detailed medical history and medications (using well-developed methods) to account for these potential confounding variables. Specific attention will be paid to beta blockers and steroids and cardiovascular co-morbidities as potential modifiers, given their known influence on our primary cardio-metabolic outcomes. As these factors can be initiated or changed during the trial, we will incorporate them as time-varying covariates within our proposed LMM approach. Our initial strategy for these changes will be to add an indicator (time-varying) for whether their medication or co-morbidity was initiated, increased, or decreased. Since we have identified and controlled the type I error for our two primary assessments (via the single, simultaneous two-degree-of-freedom F-test), no further multiplicity adjustments for our measures specified a priori as secondary will be used. We will fully disclose the measures tested and their a priori classification as primary versus secondary when reporting our findings in the literature to control the type I error.

Analyses will be performed using statistical software such as SAS and R. LMMs will be assessed by residual analysis (predicted vs. residual plots, q-q plots, etc.), visual inspection of residuals, and comparison of estimated standard deviations across groups (for homogeneity of variance). Our model assessment will use alternative strategies (transformation, bootstrapping, etc.) if indicated.

2.9.3 Aim 3

The goal of Aim 3 is to test the effect of the LEAP! Rx Program on chronic disease risk factors, including insulin resistance (HOMA2), body composition, and lipids (total cholesterol, LDL / HDL). We hypothesize the LEAP! Rx Program will positively affect an individual's metabolic profile with measurable benefits in insulin resistance, reduced fat mass / increased lean mass, and lipid status.

The secondary study measures for aim 3 include HOMA2, lean mass, fat mass, total cholesterol, HDL, and LDL at baseline, 12-, and 52-week time points; thus, similar analysis methods as in aim 1 will be used (LMMs). Assessments for these measures will examine change from baseline to 12 weeks and whether any benefits will be sustained at 52 weeks via a single statistical test. These two degree-of-freedom tests will better control the operating characteristics study-wide. For each measure, we will assess the corresponding LMM by residual analysis (predicted vs. residual plots, q-q plots, etc.), visually inspect residuals, and compare estimated standard deviations across groups (for homogeneity of variance). These assessments will use alternative strategies (transformation, bootstrapping, etc.) as indicated.
